# Prevalence and risk factors of pre-neoplastic polyps in patients with ulcerative colits: a cross-sectional retrospective study

**DOI:** 10.31744/einstein_journal/2026AO1418

**Published:** 2026-07-22

**Authors:** Flávia Carolina Oliveira, Maria de Lourdes Setsuko Ayrizono, Glaucia Fernanda Soares Ruppert Reis, Cristiane Kibune Nagasako

**Affiliations:** 1 Universidade Estadual de Campinas Faculdade de Ciências Médicas Department of Internal Medicine Campinas SP Brazil Graduate Program in Clinical Medicine, Department of Internal Medicine, Faculdade de Ciências Médicas, Universidade Estadual de Campinas, Campinas, SP, Brazil.; 2 Universidade Estadual de Campinas Faculdade de Ciências Médicas Department of Surgery, Coloproctology Unit Campinas SP Brazil Department of Surgery, Coloproctology Unit, Faculdade de Ciências Médicas, Universidade Estadual de Campinas, Campinas, SP, Brazil.; 3 Universidade de Campinas Faculdade de Ciências Médicas e Gastrocentro Department of Internal Medicine Campinas SP Brazil Gastroenterology Unit, Department of Internal Medicine, Faculdade de Ciências Médicas e Gastrocentro, Universidade de Campinas, Campinas, SP, Brazil.

**Keywords:** Colitis, ulcerative, Intestinal polyps, Colonic polyps, Adenomatous polyps, Colorectal neoplasms, Risk factors, Prevalence

## Abstract

**Objective:**

To Describe the prevalence of adenomatous and serrated polyps in ulcerative colitis and investigate risk factors associated with the development and recurrence of preneoplastic polyps.

**Methods:**

In this cross-sectional study, colonoscopy findings and clinical data from patients diagnosed with ulcerative colitis who were treated at a tertiary care center were analyzed.

**Results:**

A total of 240 patients were included (56.3% female, 79% with left-sided or extensive colitis, and 7.5% diagnosed with primary sclerosing cholangitis). The prevalence of adenomatous and serrated polyps was 16.2% (39/240). Age >41 (OR=2.85, 95%CI=1.4 - 5.8; p=0.0125), older age at the first colonoscopy (OR=1.04, 95%CI=1.01 - 1.06; p=0.0015), and higher body mass index (OR=1.12, 95% CI=1.0 - 1.1; p=0.0042) were identified as risk factors for polyps development (p<0.05). Regarding recurrence, 11 out of 39 patients presented with polyps on subsequent colonoscopies, with a mean time to recurrence of 47.5 months. No factors were found to be associated with polyp recurrence.

**Conclusion:**

The prevalence of adenomatous and serrated polyps in patients with ulcerative colitis appeared to be lower than that reported in the general population.

## Highlights

The role of adenomatous and serrated polyps in ulcerative colitis-related colorectal cancer remains unclear.

Data regarding adenomatous and serrated polyps in ulcerative colitis remain scarce.

Preneoplastic polyps were identified in 16.2% of patients with ulcerative colitis.

Age > 41 years and higher body mass index were associated with an increased risk of polyps.

## In Brief

Ulcerative colitis is associated with an increased risk of colorectal cancer. However, the role of sporadic precursor lesions, including adenomatous and serrated polyps, in ulcerative colitis -associated carcinogenesis remains unclear. This study evaluated the prevalence of these lesions and the risk factors associated with their development in patients with ulcerative colitis.

## Graphical abstract

**Figure:**
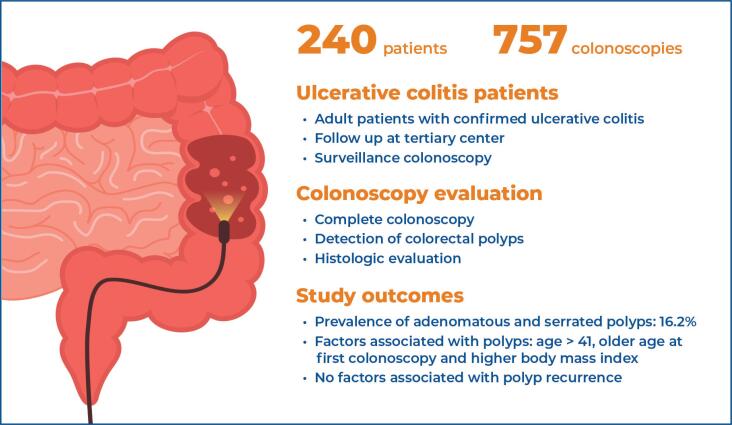

## INTRODUCTION

Patients with ulcerative colitis (UC) have an increased risk of developing colorectal cancer (CRC), estimated to be 2 to 5-fold higher compared to the general population in the same age group. This risk increases with disease duration, extent of colitis, family history of inflammatory bowel disease (IBD), presence of primary sclerosing cholangitis (PSC), and the degree of endoscopic and microscopic inflammation.^(1,2)^

About 70% of sporadic CRC cases (non-hereditary and unrelated to IBD) develop from the adenoma-carcinoma pathway, whereas 30% are associated with the serrated pathway.^(3)^ Dysplastic lesions in IBD are detected in up to 20% of surveillance colonoscopies and can be classified as invisible (dysplasia detected in random biopsies, not endoscopically delimited) or visible, the latter being more prevalent, according to recent studies.^(2,4)^ In the context of UC, the role of adenomatous and serrated polyps in the development of intestinal neoplasia remains under investigation. Despite previous analyses suggesting a lower frequency of preneoplastic lesions in these patients compared with the general population, the prevalence of such lesions in patients with UC remains unclear.^(5,6)^

## OBJECTIVE

Therefore, the primary aim of this study was to describe the prevalence of adenomatous and serrated polyps in patients with ulcerative colitis and to assess the main risk factors associated with the development and recurrence of preneoplastic polyps.

## METHODS

### Patient selection and data collection

In this cross-sectional study, patients with a confirmed diagnosis of UC (based on clinical, endoscopic, and histological criteria) from the Gastroenterology and Coloproctology outpatient clinics of *Universidade Estadual de Campinas* (Unicamp) who were referred for dysplasia and CRC surveillance colonoscopy were selected. Colonoscopies performed from January 2010 to February 2020 using a high-definition device (Olympus 170) were analyzed. The study evaluated the number of colonoscopies performed, the degree of endoscopic activity (graded according to the Mayo Score), and the presence of serrated and adenomatous polyps.

Adenomatous polyps were categorized according to histology (tubular, villous, or tubulovillous), degree of dysplasia (low and high-grade), and location (right, left colon, or rectum). The number and size of polyps were recorded, with size categories defined ≤5mm, 6-9mm, 10-19mm, or ≥20mm. Polyp morphology was classified according to the Paris endoscopic classification: 0-I (polypoid, including sessile [Is] and pedunculated [Ip] lesions) or non-polypoid (subdivided into slightly elevated [IIa], flat [IIb], slightly depressed [IIc], and excavated [III]. Factors associated with polyp recurrence were also assessed, including inflammatory activity, number of polyps, histology, endoscopic appearance, size, and location.

The clinical data evaluated included sex, ethnicity, age at diagnosis, age at the first colonoscopy, extent of intestinal involvement (according to the Montreal classification: E1 Proctitis, E2 left-side colitis and E3 pancolitis), body mass index (BMI), diagnosis of PSC, smoking status, family history of IBD, current treatment during the examination (isolated or combined use of aminosalicylates, azathioprine and biologics) and need of colorectal surgery prior the beginning of the study. Patients without a confirmed diagnosis of UC or without follow-up colonoscopy data available in the hospital's database were excluded, as were colonoscopies with inadequate bowel preparation (Boston scale score <6) and incomplete examinations (not reaching the cecum).

### Ethical considerations

This study was approved by an Ethics and Research Committee of the *Universidade Estadual de Campinas* (CAAE: 46691321.0.0000.5404; #4.781.031).

### Statistical analysis

The prevalence of adenomatous and serrated polyps was expressed as a percentage. Categorical variables were evaluated using the χ^2^ test and, when necessary, Fisher's exact test. Numeric variables were analyzed using the Mann-Whitney test. Logistic regression analysis was performed to assess risk factors associated with polyp development. Variables for the multivariable models were selected using the stepwise method. A p<0.05 was considered statistically significant.

## RESULTS

### Clinical features of patients

A total of 240 of the 300 patients eligible for the study were included. The median follow-up period in this series was 5 years. One hundred thirty-five (56.3%) were female, with a median age at first colonoscopy of 44±15 years. According to the Montreal classification for age at disease onset, 10 (4.1%) were aged ≤16 years, 151 (62.9%) were between 17 and 40 years, and 79 (32.9%) were older than 40 years.

The median BMI was 24.4±4.72 kg/m^2^, and 45% of the patients were overweight (32%) or obese (12.7%).

As for intestinal involvement, 115/240 patients (47.9%) had pancolitis, 75/240 (31.3%) had left-sided colitis, and 50/240 (20.8%) had ulcerative proctitis. Endoscopic remission was observed in 55.4% of the patients (Mayo Score 0 or 1). The median age at detection of the first polyp was 54.3±12.2 years. Eight patients underwent surgical treatment during follow-up (4 due to high-grade dysplasia, 3 due to clinical refractoriness, and 1 due to acute severe UC). All patients with high-grade dysplasia had active pancolitis on endoscopy: three had multifocal high-grade dysplasia in areas of mucosal irregularity (one of whom was diagnosed with colorectal neoplasia on histological examination of the surgical specimen), and one patient had invisible high-grade dysplasia. The clinical characteristics of the patients are summarized in table 1.

**Table 1:** Clinical characteristics of the sample

Female / Male		135 (56.3) /105 (43.7)
Age at diagnosis (years)		34±14.3
Age at first colonoscopy (years)		44±15
Age at first polyp detection (years)		54.3±12.2
Montreal classification		
	E1	50 (20.8)
	E2	75 (31.3)
	E3	115 (47.9)
Endoscopic remission (Mayo 0/1)		133 (55.4)
Surgery		8 (3.3)
High-grade dysplasia (HGD)		4/8 (50)
CIinical Intractability		3/8 (37.5)
Toxic Megacolon		1/8 (12.5)
Ethnicity		
	White	179 (74.6)
	Black	15 (6.2)
	Brown (mixed ethnicities)	46 (19.2)
Primary sclerosing cholangitis (PSC)		18 (7.5)
BMI		24.4±4.72
Familial history of IBD		11/107 (10.3)
Smoking history		21/115 (18.3)
Treatment		
	5- aminosalicylates	178/232 (76.7)
	Azathioprine	101/232 (43.5)
	Immunobiologicals	95/232 (40.9)

Expressed as n (%) or median±standard deviation.

HGD: high-grade dysplasia; PSC: primary sclerosing cholangitis; BMI: body mass index; IBD: inflammatory bowel disease.

When comparing groups with and without endoscopic activity (Mayo score 0 or 1), patients with endoscopic activity were younger (median age at diagnosis: 32±13.8 years *versus* 39±14.3 years p=0.002) and underwent their first colonoscopy at an earlier age than patients in endoscopic remission (median age at first colonoscopy 41.1±14.9 years *versus* 47.2±14.5 years, p=0.002). Additionally, immunobiologicals were used by 56% of patients withendoscopic activity compared with 39% of those in remission (p<0.001).

### Endoscopic findings

Of the 757 colonoscopies analyzed, 53 (7%) were excluded due to inadequate bowel preparation and/or incomplete examination. Therefore, 704 colonoscopies were eligible for the study. The number of colonoscopies per patient ranged from 1 to 7.

A total of 87 polyps (82 adenomatous and five serrated) were detected in 39 patients (16.2%). Among these patients, 30 (77%) were in endoscopic remission, whereas 9 (23%) had endoscopic activity. The characteristics of the adenomatous polyps are detailed in table 2. The prevalence of adenomatous polyps was 15.4% (37/240), while serrated polyps were identified in 1.6% (4/240) of the patients. Among the serrated polyps, three patients had sessile serrated adenomas, and one had a traditional serrated adenoma with low-grade dysplasia. Patients with polyps were older than those without polyps (median age 42±13.9 *versus* 33±14.3 years, p=0.0078) and underwent their first colonoscopy at an older age (median age at first colonoscopy: 53.1±12.5 *versus* 42.8±15.1 years, p=0.0006). Additionally, BMI was higher in patients with polyps (25.7±5.2kg/m² *versus* 24.1±4.5kg/m², p=0.0093). A comparison between patients with and without polyps is shown in table 3.

**Table 2:** Characteristics of adenomas expressed as n (%)

Adenoma		n=82
Histology		
	Tubular	68 (82.9)
	Villous	0 (0)
	Tubulovillous	14 (17.1)
Grade of dysplasia		
	Low grade	72 (87.8)
	High grade	10 (12.2)
Paris classification		
	Is	79 (96.3)
	Ip	3 (3.6)
Location		
	Right colon	50 (61)
	Left colon	21 (25.6)
	Rectum	11 (13.4)
Size		
	≤5mm	56 (68.3)
	6-9mm	15 (18.3)
	10-19mm	7 (8.5)
	≥20mm	4 (4.9)

Is: including sessile; Ip: including pedunculated.

**Table 3:** Patients with and without polyps

		Polyps n=39	No polyps n=201	p value
Age at diagnosis (years)		42±13.9	33±14.3	0.0078*
Age at first colonoscopy (years)		53.1±12.5	42.8±15.1	0.0006*
Female / Male		20 (51.3) / 19 (48.7)	115 (57.2) / 86 (42.8)	0.4944
Ethnicity				
	White	29 (74.4)	150 (74.6)	0.4621
	Black	4 (10.2)	11 (5.5)	
	Brown (mixed ethnicities)	6 (15.4)	40 (19.9)	
BMI		25.7±5.2	24.1±4,5	0.0093*
PSC		3 (7.7)	15 (7.5)	1.0000
Montreal classification				
	E1	12 (30.8)	38 (18.9)	0.1592
	E2	13 (33.3)	62 (30.8)	
	E3	14 (35.9)	101 (50.2)	
Familial history of IBD		1/16 (6.3)	10/91 (11)	1.0000
Smoking history		2/17 (11.8)	19/98 (19.4)	0.7343
Endoscopic remission		30 (77%)	104 (51.7)	0.0020*

* p<0.05.

Data are expressed as n (%) or median±standard deviation.

BMI: body mass index; PSC: primary sclerosing cholangitis; IBD: inflammatory bowel disease.

On multivariable analysis, the presence of adenomatous polyps was associated with age >41 years (OR=2.85, 95%CI=1.4 - 5.8; p=0.0125), older age at the first colonoscopy (OR=1.04, 95%CI=1.01 - 1.06; p=0.0015), and higher BMI (OR=1.12, 95%CI =1.0 - 1.1; p=0.0042). Family history of inflammatory bowel disease, smoking status, ethnicity, sex, and concomitant PSC were not associated with the presence of polyps.

Regarding polyp recurrence, 11/39 patients presented with polyps on subsequent colonoscopies, with a mean time to recurrence of 47.5 months. Inflammatory activity, histology, endoscopic appearance, location, size >6mm, and number of polyps did not influence recurrence.

## DISCUSSION

In this study, the prevalence of serrated and adenomatous polyps in patients with UC was 16.2%, which was higher than that reported in other countries. A study conducted in London that analyzed 150 patients with UC, with a mean age of 52 years, reported a prevalence of adenomatous polyps of 4%, lower than the 12% observed in the general population (p<0.01).^(7)^ In Turkey, the reported prevalence was 2.2% (mean age 35.9±13.5 years).^(8)^ This discrepancy may be related to ethnic and geographic differences.

Regarding serrated polyps, our analysis identified a prevalence of 1.6%. This finding is consistent with that of a recent study evaluating 2,035 colonoscopies in patients with UC, which reported a prevalence of 1.8%.^(9)^ In a retrospective study conducted in our region, the prevalence of serrated polyps in the general population (mean age 61.3±11.5 years) was 0.6%.^(10)^ Data regarding the prevalence of adenomatous polyps in the Brazilian population without IBD are scarce. CRC screening programs in which colonoscopy was performed in individuals older than 50 years with a positive fecal immunochemical test (FIT) have shown variable results. Guimarães et al. reported a high adenoma detection rate (61%) in individuals with positive FIT results and a mean age of 57±4.45 years.^(11)^ In a prospective study conducted at our institution, the prevalence of polyps among individuals (mean age 55.07±5.49 years) with positive FIT results who underwent colonoscopy was 37.6% in the first year and 30.3% in the second year of implementation of a CRC prevention program.^(12)^

These numbers are higher than those observed in our analysis, which found a prevalence of 16.2% in individuals with a median age at first colonoscopy of 44 years. Other authors have also reported a low prevalence of adenomas in patients with UC. In a retrospective cohort of 403 patients with UC older than 40 years (82.6% with left or extensive colitis) and a mean follow-up of 6.3 years, the prevalence of colorectal adenomas was 11.3%.^(13)^ In another retrospective analysis, the prevalence of adenomatous polyps at index colonoscopy in patients with UC older than 50 years was lower than that observed in the Control Group (13/206 [6.3%] *versus* 162/624 [25.9%]).^(14)^ Some hypotheses have been proposed to explain this apparently low prevalence of adenomatous polyps in individuals with UC, including the protective anti-inflammatory effect of aminosalicylates, increased immune reactivity in the colon associated with UC leading to elimination of adenoma precursor cells, and the difficulty in detecting polyps in inflamed mucosa.^(14,15)^

In our study, patients in endoscopy remission (Mayo Score 0/1) had a higher prevalence of polyps (77% of patients in remission *versus* 23% of patients with endoscopic activity), corroborating the hypothesis that polyps are more difficult to detect in the presence of inflammation and highlighting the importance of performing surveillance colonoscopy during remission.

Age (at diagnosis and at the first colonoscopy) and BMI were identified as factors associated with polyp development. The relatively late mean age at the first colonoscopy (44.1±15 years) may reflect the fact that most patients in the sample developed their first symptoms between 17 and 40 years of age (62.9%).

We observed that age >41 years increased the risk of developing polyps by 2.8-fold. In the general population, it is well established that the prevalence of preneoplastic polyps increases with age. Since 2021, the American College of Gastroenterology has recommended initiating CRC screening at 45 years of age.^(3)^ Previous studies have reported that individuals with higher BMI and those with metabolic syndrome are at increased risk of developing adenomatous and non-adenomatous polyps.^(16)^ In our analysis, higher BMI was associated with an 11.2-increase in the risk of developing polyps. A possible explanation is the contribution of the inflammatory state associated with overweight and obesity (including increased tumor necrosis factor and interleukin-6 levels) to the development of adenomatous polyps and CRC.^(16)^ A Chinese study evaluating risk factors for polyp development in the general population reported that individuals with a BMI ≥25kg/m^2^ and those with metabolic syndrome had an increased risk of developing adenomatous and non-adenomatous polyps (p<0.001).^(16)^ Eleven of the 39 patients presented with polyps on subsequent colonoscopies. Factors such as inflammatory activity, number of polyps, histology, endoscopic appearance, size >6mm, and polyp location were not associated with recurrence. Some authors have reported active inflammation, the number of polyps (≥3), high-grade dysplasia, and proximal location as factors associated with polyp recurrence in the general population.^(17)^ The low recurrence prevalence limited this analysis in our study.

A single case of colorectal neoplasia occurred in a 32-year-old male patient with 15 years of disease duration, active pancolitis, PSC, and refractoriness to infliximab treatment. Colonoscopy revealed a 15 mm slightly elevated lesion in the cecum (adenoma with high-grade dysplasia on biopsy) and multifocal high-grade dysplasia on targeted biopsies of suspicious areas in the remaining colonic segments. Following proctocolectomy, histopathological analysis of the surgical specimen demonstrated a well-differentiated adenocarcinoma in the cecum measuring 17 x 10 mm, with invasion up to the muscular layer and perineural invasion, without vascular or lymphatic invasion and without lymph node metastasis. According to the 2018 ECCO guidelines, CRC surveillance should be initiated in all patients with UC after eight years from symptom onset in order to screen for neoplastic lesions, assess disease extent, and exclude dysplasia.^(18)^ The interval between subsequent colonoscopies depends on high-risk factors for neoplasia, such as pancolitis, concomitant PSC, and/or severe inflammatory activity. Patients at increased risk for CRC should undergo annual colonoscopy surveillance. ^(18)^

Younger age at diagnosis is a factor associated with greater IBD severity.^(19)^ In our analysis, patients with endoscopic activity were younger than those in remission (median age of 32 years *versus* 39 years). They also had more severe diseases, as 56% of patients with endoscopic activity required immunobiological therapy, compared with 39% of patients in remission (p<0.001).

Our study has some limitations. This was a retrospective, single-center study. In our analysis, a Control Group of individuals without ulcerative colitis matched by age was not included, which limited the ability to determine the true difference in polyp prevalence between patients with and without UC. During the review of medical records, the lack of some data, such as smoking history and a family history of IBD, limited the assessment of their impact on polyp development. Furthermore, endoscopic disease activity (Mayo score >1) was present in 44.6% of patients, which may have impaired the detection of adenomatous and serrated polyps in inflamed mucosa.

## CONCLUSION

The prevalence of adenomatous and serrated polyps in patients with ulcerative colitis appeared to be lower than that reported in the general population. No factors associated with polyp recurrence were identified in our sample.

## Data Availability

The data underlying this study are included in the manuscript.
